# Donor variability may mask dimethyl fumarate’s effects on nuclear factor E2-related factor 2 in human peripheral blood mononuclear cells

**DOI:** 10.1186/s13104-017-2862-8

**Published:** 2017-11-02

**Authors:** Sarah E. Fiedler, Amelia R. Kerns, Catherine Tsang, Haley N. Love, Sonemany Salinthone

**Affiliations:** 1 VA Portland Health Care System, Research and Development Service, 3710 SW US Veterans’ Hospital Rd., Mail Code R&D8, Portland, OR 97239 USA; 2 Department of Neurology, Oregon Health & Sciences University, 3181 SW Sam Jackson Park Rd., Portland, OR 97239 USA

**Keywords:** Dimethyl fumarate, Nrf2, Peripheral blood mononuclear cell, HO-1, Cyclic AMP

## Abstract

**Objective:**

Dimethyl fumarate (DMF) is an anti-inflammatory and antioxidant drug used to treat multiple sclerosis, but its mechanism(s) of action are not fully understood. In central nervous system (CNS) cells, DMF activates nuclear factor E2-related factor 2 (Nrf2), perhaps ameliorating oxidative stress-induced damage. However, it is not known whether DMF also activates Nrf2 in peripheral immune cells, which are known to participate in CNS demyelination. We conducted a single observation study to determine whether DMF can activate Nrf2 in peripheral immune cells in vitro.

**Results:**

We performed enzyme-linked immunosorbent assays to measure Nrf2 activation in nuclear extracts of human peripheral blood mononuclear cells treated with DMF at time points from 0 to 6 h, initially determining that DMF did not activate Nrf2, and that the mechanism(s) of action of DMF may thus differ in the periphery compared to the CNS. However, further analyses of our data suggest that high T_max_ variability is masking Nrf2 activation in individual donors. Additionally, there may be sub-populations of responders, perhaps related to genetic polymorphisms in Nrf2.

**Electronic supplementary material:**

The online version of this article (10.1186/s13104-017-2862-8) contains supplementary material, which is available to authorized users.

## Introduction

Dimethyl fumarate (DMF) is an anti-inflammatory and antioxidant drug approved for the treatment of relapsing–remitting multiple sclerosis (MS). Treatment with DMF leads to significant reductions in relapse rates, progression of disability, and active brain lesions [1]. The mechanisms of action (MOA) of DMF are not entirely understood, but are believed to involve therapeutic effects on immune dysregulation and oxidative stress that lead to the pathology of MS. Nuclear factor E2-related factor 2 (Nrf2) is the principal regulator of the phase II cellular antioxidant response, and there is extensive evidence that DMF alters Nrf2 expression and activation in vitro in central nervous system (CNS) cells [2].

While MS is a neuro-inflammatory disease, MS likely begins with the peripheral immune system inappropriately activated in response to an environmental antigen, and pathogenesis is immune cell mediated [3, 4]. Peripheral immune cells, including monocytes which migrate into the CNS to become macrophages, contribute to disease progression. Active MS lesions are infiltrated by large numbers of peripherally derived-macrophages that contain myelin debris, indicating that they are responsible for demyelination [5–7]. Taken together, we hypothesize that given its efficacy in reducing active brain lesions, orally administered DMF is likely to have an MOA that includes effects on peripherally-derived macrophages, and that it involves activation of Nrf2. However, direct evidence that DMF acts via Nrf2 in peripheral immune cells is, to our knowledge, absent. Herein, we conducted a single observation study to determine whether DMF can activate Nrf2 in peripheral blood mononuclear cells (PBMCs).

## Main text

### Materials and methods

#### Cell stimulation and sample processing

Apheresis products were purchased from KeyBiologics (Memphis, TN). The company followed IRB regulations for consenting of human donors. PBMCs were isolated and cryopreserved as previously described [8]. PBMCs were quick thawed and washed in RPMI (Gibco by Thermo Fisher, Waltham, MA) prior to manual counting with Trypan Blue (Sigma-Aldrich, St. Louis, MO) viability assessment. At least 3.2 × 10^6^ viable cells were used per treatment. In initial time-course experiments, cells were resuspended in serum-free RPMI, then left untreated, treated with vehicle (ethanol), or treated with 200 µg/ml DMF (Sigma-Aldrich, St. Louis, MO) for 30 min to 6 h at 37 °C/5% CO_2_. Nuclear extraction was performed according to TransAM Nrf2 kit protocol (Active Motif, Carlsbad, CA). Protein concentration for each sample was determined using a bicinchoninic acid (BCA) assay kit, according to manufacturer’s protocol (Pierce, Rockford, IL).

In a second set of experiments, we used a commercial nuclear extraction kit according to manufacturer’s protocol (Cayman Chemical, Ann Arbor, MI), added 2.5% FBS (final concentration; Gibco, Waltham, MA) to the samples 15 min after DMF addition in order to improve cell survival (also added to UNT and VEH), reduced the number of incubation time points to 2 and 4 h (those most likely to show activation based on literature), and added UNT controls for both time points (formerly untreated samples were representative of the longest time point in each assay).

#### Enzyme-linked immunosorbent assays (ELISAs)

To measure Nrf2 activation, we used the TransAM Nrf2 kit, a plate based ELISA kit on which oligonucleotide containing the ARE (antioxidant response element) consensus binding site has been immobilized. The active form of Nrf2 binds to this site, and the detection antibody recognizes an epitope on Nrf2 upon DNA binding. Five micrograms of nuclear extract per sample was assayed. Positive control was Nrf2-transfected COS-7 nuclear extract. Assays were performed according to manufacturer’s protocols in duplicate or triplicate, and plates were read using a SpectraMax M2 microplate reader (Molecular Devices, Sunnyvale, CA).

To measure cyclic AMP (cAMP) production as a test of DMF efficacy, 2 × 10^6^ viable PBMCs (per treatment) were treated for 5 min with 50, 100 and 200 µg/ml of DMF, or treated with 5 µl of vehicle control (ethanol, volume equal to 200 µg/ml DMF dose). Samples were processed and cAMP assay performed according to manufacturer’s protocol (Enzo Life Sciences, Farmingdale, NY).

#### Data and statistical analyses

Values for each donor were determined as an average of the replicates, which were generated from internal 4 parameter logistic standard curves using Softmax software (Molecular Devices) for cAMP assays, or absorbance at 450 nm for Nrf2 assays. These values were used to determine the arithmetic mean, plus or minus the standard error of the mean, of each condition. Treatments and controls were tested for significant differences in Microsoft Excel 2010 using one-way ANOVAs and Student’s t-tests.

### Results

**Fig. 1:**
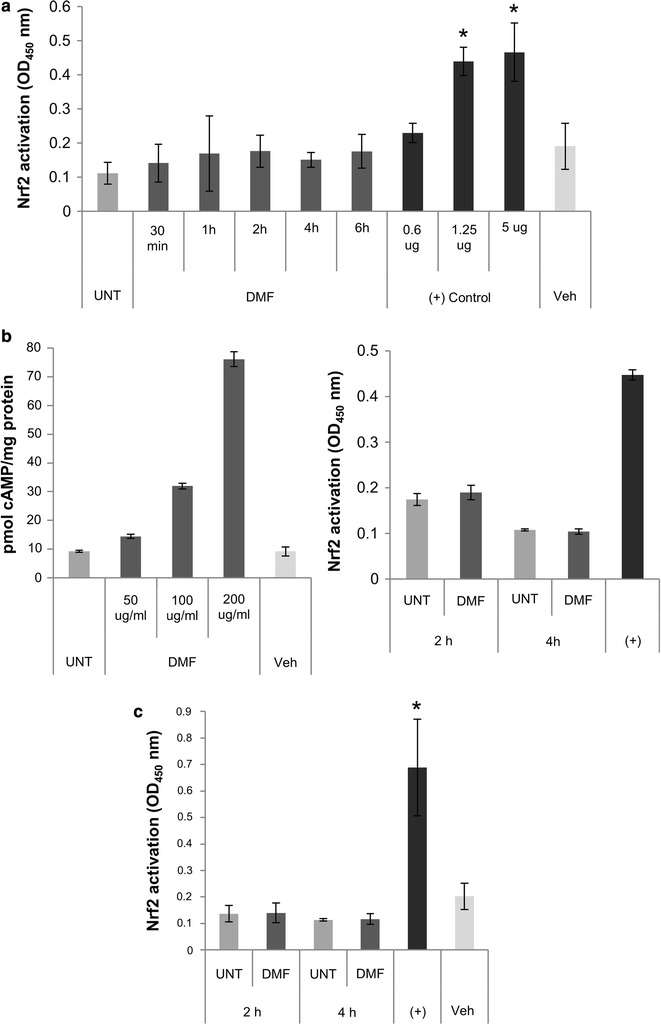
Dimethyl fumarate does not appear to activate Nrf2 in time course experiments with human peripheral blood mononuclear cells (PBMCs). **a** PBMCs were left untreated (UNT), treated with vehicle (Veh), or treated with 200 µg/ml DMF for the time indicated. Nuclear extracts were isolated and 5 µg extract per well was used for TransAM Nrf2 ELISAs (Active Motif) performed in duplicate or triplicate. Positive (+) control Nrf2-transfected COS-7 nuclear extract was added to wells in the amounts indicated. Allogeneic donors for each time point: 30 min and 1 h, N = 4 each, 2 h N = 6, 4 h N = 5, and 6 h N = 3. *Indicates p < 0.05 compared to UNT. **b** PBMCs from a single donor were used for cAMP (left panel, Enzo Life Sciences) and Nrf2 ELISAs (right panel). For cAMP assay, cells were left untreated (UNT), treated with vehicle (Veh, ethanol), or treated with DMF at the indicated concentrations for 5 min; For Nrf2 assay, cells were treated with 200 µg/ml DMF for the time indicated. **c** PBMCs were left untreated (UNT) or treated with 200 µg/ml DMF for the time indicated, with the addition of FBS to 2.5% after the first 15 min of DMF treatment. Nuclear extracts were prepared using a commercially available kit (Cayman Chemical) and assayed for Nrf2 activation as above, performed in duplicate or triplicate. Five micrograms of positive control (+) Nrf2-transfected COS-7 nuclear extract was used. Allogeneic donors for each time point: 2 h UNT, 2 h DMF and 4 h DMF N = 6 each; 4 h UNT N = 3. *Indicates p < 0.05 compared to 2 h UNT

Treatment of PBMCs with DMF did not appear to result in Nrf2 activation (Fig. 1a). To ensure that the DMF stocks we were using were active, we performed parallel experiments where PBMCs from the same donor were treated with varying concentrations of DMF for cAMP and Nrf2 assays. We previously demonstrated that DMF stimulates cAMP production in PBMCs [8]. Here, DMF stimulated cAMP production in a similar manner (Fig. 1b, left panel), but did not appear to activate Nrf2 in this donor (Fig. 1b, right panel).

In order to eliminate technical difficulties as the reason for failure to achieve Nrf2 activation, we analyzed cell survival post-treatment and added varying concentrations of FBS to optimize survival. UNT and DMF treated samples did not differ significantly in their survival regardless of FBS presence or concentration, but 2.5% FBS resulted in slightly higher survival for both compared to serum-free RPMI at 2 and 4 h (data not shown). We also varied the amount of protein used in ELISAs (1–10 µg, the max recommended), and attempted stimulation with monomethyl fumarate (active metabolite of DMF). None of these conditions resulted in consistent Nrf2 activation (data not shown).

**Fig. 2:**
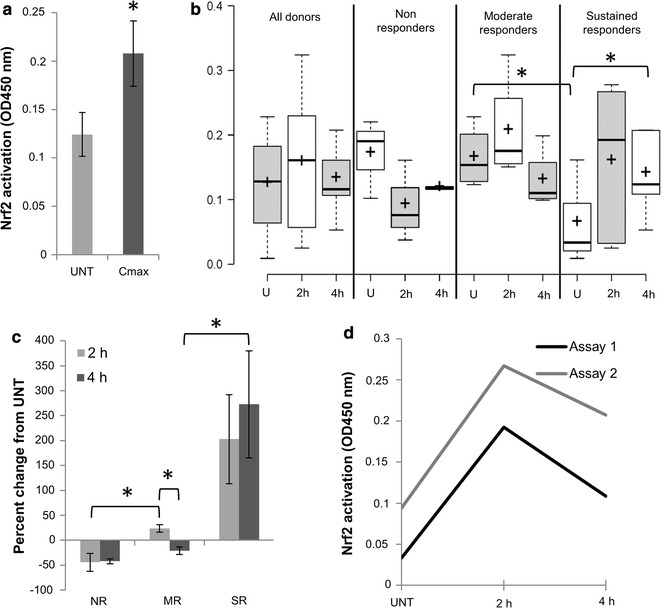
Dimethyl fumarate has donor-dependent effects on Nrf2 in human peripheral blood mononuclear cells (PBMCs). Analyses presented in this figure are derived from the data presented in Fig. 1 experiments, combining donors from Fig. 1a, c. **a** Untreated (UNT) values are compared to the maximum Nrf2 activation (OD450) for all donors (C_max_). n = 12, 11 allogeneic. **b** Donor values for UNT, 2 h DMF and 4 h DMF were analyzed in aggregate (all donors), or broken into groups based on Nrf2 response to DMF treatment. Non-responders: Nrf2 decreases with DMF treatment. Moderate responders: Nrf2 increases modestly at 2 h but drops below UNT by 4 h. Sustained responders: Nrf2 robustly activated at 2 h, remains above UNT at 4 h. Center lines show the medians; box limits indicate the 25th and 75th percentiles as determined by R software; whiskers extend 1.5 times the interquartile range from the 25th and 75th percentiles, outliers are represented by dots; crosses represent sample means. n = 12, 12, 11, 3, 3, 2, 4, 4, 4, 5, 5, 5 sample points. This graph was generated by BoxPlotR: a web-tool for generation of box plots. **c** Graph representing the direction and magnitude of Nrf2 activation status with DMF treatment. Percent change was calculated by subtracting UNT values from the value at the time point at which Nrf2 showed the largest change in activation from compared to UNT (whether that was an increase or decrease) for each donor. *NR* non-responder, *MR* moderate responder, *SR* sustained responder. **d** Nrf2 results for the same donor repeated in both the Fig. 1a, c experiments demonstrate consistency of donor response to DMF. This donor is a sustained responder. In all panels, *indicates p < 0.05 by Student’s t-test. ANOVA was used prior to t-tests in B and C

Finally, to eliminate our extraction method as the reason for our results, we switched to a commercial nuclear extraction kit and repeated ELISAs at 2 and 4 h plus 2.5% FBS (Fig. 1c). These data appeared to confirm that Nrf2 is not activated by DMF in human PBMCs. However, when we analyzed our data set as a whole, including all donors from both our initial time courses (1a) and our optimized time courses (1c), we noticed that several donors showed an elevation in Nrf2, but there was high variability in the time point at which the stimulation occurred (T_max,_ Table 1). We thus decided to compare UNT values versus maximum Nrf2 assay values (C_max_), regardless of time point (Fig. 2a). In the analysis, the difference in Nrf2 activation between UNT and DMF treated cells was significant (p < 0.05), so it is possible that Nrf2 is being stimulated by DMF, just with a highly variable time course. It should be noted that for three of the donors C_max_ were UNT values, meaning they exhibited no Nrf2 stimulation at any time point. Continuing our examination of individual donor responses, we observed three distinct patterns; in “non-responders,” Nrf2 is slightly decreased with DMF treatment, in “moderate responders,” Nrf2 is increased modestly at 2 h but drops below UNT by 4 h, and in “sustained responders,” Nrf2 is robustly activated at 2 h, and remains above UNT at 4 h (Fig. 2b, c). Within our combined data set, we had one donor that was analyzed in experiments for both Fig. 1a and c, meaning that we could compare whether donor response type is consistent. Figure 2d indicates that this donor, who appeared to be a sustained responder in our initial experiments, responded in the same manner in our optimized time courses.

**Table 1:** T_max_ distribution

Time of peak Nrf2 activation (T_max_, in h)	Number of donors
0	3
0.5	1
1	1
2	5^a^
4	2

^a^4 allogeneic donors, 1 donor repeated

### Discussion

The MOA(s) of DMF are still under investigation, but activation of Nrf2 and its downstream effectors in the CNS and periphery has been a primary focus. Recently however, DMF treatment was shown to be equally protective in the mouse model of MS in wild type and Nrf2^−/−^ mice, and protection was associated with changes in T cell, monocyte and B-cell phenotypes, raising the possibility that Nrf2 is not essential for DMFs therapeutic actions on peripheral immune cells [9]. Studies of DMF treated patients also show alterations in the phenotypes and ratios of these cell types [10–12]. In apparent contrast to these data, Gopal et al. published a paper using PBMCs entitled, “Evidence of activation of the Nrf2 pathway in multiple sclerosis patients treated with delayed-release dimethyl fumarate”, however, this study did not directly examine activation of Nrf2, but rather examined induction of downstream targets NAD(P)H quinone dehydrogenase-1 (NQO1) and heme-oxygenase-1 (HO-1) at time points from 0 to 4 h [13].

In addition to its widely reported activation of Nrf2 in CNS cells, in vitro DMF treatment of peripheral immune cells has been shown to affect NF-κB activation and production of a variety of inflammatory mediators; we have previously demonstrated that DMF stimulates production of the immunomodulator cAMP via the prostaglandin E2 receptor in PBMCs, but only at relatively high concentrations of DMF [2, 8]. It is possible that the MOA of DMF is primarily dependent on activating Nrf2 in the CNS to ameliorate oxidative stress, while its MOA in the periphery involves other immunomodulatory pathways, some of which may have effectors in common with the Nrf2 pathway; the aforementioned HO-1, for example, which can be induced by both cAMP and Nrf2-dependent pathways [13–15]. Of particular interest in this case, Gopal’s above mentioned study showed induction of HO-1 only at high concentrations of DMF, very similar to the pattern of cAMP induction we previously demonstrated and present as control data in Fig. 1b [8, 13]. cAMP is not the sole mediator of DMF’s therapeutic efficacy in the periphery, but we present it as a potential part of the MOA of DMF in MS, which may be either Nrf2-independent or additive/synergistic with Nrf2.

Our individual donor analyses suggest that DMF activation of Nrf2 in PBMCs is highly variable—with subgroups of responders that when aggregated across a time course mask activation. This kind of variability is to be expected in clinical trials since there are many factors in how an oral drug is taken up by patients, but is less expected in in vitro studies. These differences may be due to single nucleotide polymorphisms (SNPs) in Nrf2. There are at least nine (SNPs) in humans; two are in the promoter region of Nrf2 and reduce transcriptional activity. These latter two plus one additional SNP have been associated with variable Nrf2 response to caffeine [16]. Another possibility is that there are differences in the distribution of the PBMC sub-populations between donors that affect responses due to differential cell type responses. It is interesting to note that sustained responders have significantly lower UNT values than moderate responders (and comparison to non-responder UNT is near significance at p = 0.058, Fig. 2b), and in fact the “robust activation” seen in sustained responders simply brings their Nrf2 activation levels near the UNT levels in the other two groups. The biological/clinical meaning of this difference is unknown.

In future studies, it would be of interest to determine whether specific SNPs are associated with Nrf2 response in PBMCs. If effects on peripheral immune cells are part of the MOA of DMF, determining Nrf2 genotype might be a tool to help decide who should take the drug, which is extremely expensive and has the rare (but serious) side effect of progressive multifocal leukoencephalopathy [17].

## Limitations

There were a number of limitations to this study. The DMF concentration used is relatively high, and addition of a cell type in which DMF is known to activate Nrf2 as a positive control would have strengthened this study. Also, although DMF activates Nrf2 in other cell types within the time points we examined, it is possible that in the “non-responders,” activation is either quickly transient or delayed beyond 6 h in PBMCs. Also, though cell viability was good, the slight drops seen at 2 h (non-responders) and 4 h (non and moderate responders) may contribute to the small reduction in Nrf2 activation compared to UNT. The small sample size for our proposed subgroups makes these results far more indicative than conclusive, though it provides an interesting avenue for analyzing future experiments involving DMF responses. Finally, only healthy control cells were utilized in these studies—it will be very important to compare how cells from MS patients respond in future studies.

## Additional file

**Additional file 1.** All raw data collected and used in analyses for this manuscript, organized by figure number.

## Abbreviations

ARE: antioxidant response element
BCA: bicinchoninic acid
cAMP: cyclic AMP
CNS: central nervous system
DMF: dimethyl fumarate
ELISA: enzyme-linked immunosorbent assay
FBS: fetal bovine serum
HO-1: heme-oxygenase-1
MOA: mechanism of action
MS: multiple sclerosis
NF-κB: nuclear factor kappa B
NQO1: NAD(P)H quinone dehydrogenase 1
Nrf2: nuclear factor E2-related factor 2
PBMC: peripheral blood mononuclear cell
SNP: single nucleotide polymorphism
